# The Error-Related Negativity Predicts Self-Control Failures in Daily Life

**DOI:** 10.3389/fnhum.2020.614979

**Published:** 2021-01-27

**Authors:** Rebecca Overmeyer, Julia Berghäuser, Raoul Dieterich, Max Wolff, Thomas Goschke, Tanja Endrass

**Affiliations:** ^1^Faculty of Psychology, Technische Universität Dresden, Dresden, Germany; ^2^Department of Psychiatry and Psychotherapy, Technische Universität Dresden, Dresden, Germany; ^3^Neuroimaging Centre, Technische Universität Dresden, Dresden, Germany

**Keywords:** performance monitoring, ERN, error processing, EEG, self-control, ecological momentary assessment, daily life

## Abstract

Adaptive behavior critically depends on performance monitoring (PM), the ability to monitor action outcomes and the need to adapt behavior. PM-related brain activity has been linked to guiding decisions about whether action adaptation is warranted. The present study examined whether PM-related brain activity in a flanker task, as measured by electroencephalography (EEG), was associated with adaptive behavior in daily life. Specifically, we were interested in the employment of self-control, operationalized as self-control failures (SCFs), and measured using ecological momentary assessment. Analyses were conducted using an adaptive elastic net regression to predict SCFs from EEG in a sample of 131 participants. The model was fit using within-subject averaged response-locked EEG activity at each electrode and time point within an epoch surrounding the response. We found that higher amplitudes of the error-related negativity (ERN) were related to fewer SCFs. This suggests that lower error-related activity may relate to lower recruitment of interventive self-control in daily life. Altered cognitive control processes, like PM, have been proposed as underlying mechanisms for various mental disorders. Understanding how alterations in PM relate to regulatory control might therefore aid in delineating how these alterations contribute to different psychopathologies.

## Introduction

Adaptive behavior critically depends on monitoring response outcomes for the need to adapt behavior and the recruitment of cognitive control, a process called performance monitoring (PM) (Ullsperger et al., 2014a,b). Altered neural correlates of PM in various mental disorders associated with deficient goal-directed control, such as obsessive-compulsive disorder (OCD) and substance use disorders (SUD), indicate a link between neural measures of PM and regulatory control in daily life (Van Veen and Carter, 2002; Robbins et al., 2012; Euser et al., 2013; Endrass and Ullsperger, 2014; Gillan et al., 2017). Accordingly, self-control in daily life, as assessed *via* smartphone-based ecological momentary assessments (EMA), has been linked to error-related activity in the PM network in a functional magnetic resonance imaging (fMRI) study (Krönke et al., 2018). The aim of the current study was to establish whether PM-related brain activity as measured by the error-related negativity (ERN) predicts adaptive behavior in daily life. Given that to date most studies assessed self-control in daily life using self-report questionnaires, data on actual behavior outside the lab and the link to brain activity is still rare (de Ridder et al., 2012).

There are many models and theories on self-regulation, which focus on different levels of analysis, and which have not yet been integrated within an overarching framework (Inzlicht et al., 2020). Self-regulation is the process of ascertaining a desired goal and then taking action to move toward that goal and continuously monitoring progress and the need to adapt the behavior (Carver and Scheier, 1998). Self-regulation includes various steps, like deciding on a goal, planning how to pursue it, pursuing it, and shielding that goal from interference or competing responses (Gollwitzer, 1999; Fujita, 2011). Goal-directed behavior is thus behavior that is being performed based on the belief that a specific goal or outcome can be achieved by this behavior, and that there is a reason to seek that specific outcome (Dayan, 2009). Self-control constitutes one specific form of self-regulation, but not all forms of self-regulation include self-control (Fujita, 2011). Inzlicht et al. (2020) define self-control as targeting behavior toward a desired goal, a process which includes inhibitory as well as initiatory components and is closely connected to the implementation of behavior (de Ridder et al., 2011, 2012; Baumeister, 2014; Gillebaart, 2018). Self-control can therefore be described as the ability to change or override competing response tendencies as well as to regulate behavior, thoughts and emotions in accordance with a desired goal, and is exerted to promote desirable responses and inhibit undesirable responses or impulsive actions (de Ridder et al., 2012; Hofmann et al., 2012a). Self-regulation is a broader concept, which includes goal setting, monitoring if there is a need for the exertion of self-regulation, and implementing actions according to set goals (Baumeister and Heatherton, 1996; Carver and Scheier, 1998).

A related concept is cognitive control, which can be described as the ability to pursue goal-directed behavior, opposing otherwise more habitual or immediately compelling behaviors (Cohen, 2017). There are differing opinions on how exactly cognitive control, which in core aspects strongly resembles the concept of self-regulation, relates to self-regulation. Cognitive control is typically used as a term for employment of cognitive operations or executive functions, like inhibition, attentional shifting and working-memory updating, whereas self-regulation typically refers to adapting behavior in daily life (Hofmann et al., 2012b; Miyake and Friedman, 2012; Inzlicht et al., 2020). The broad monitoring function described in the self-regulation literature is similar to the aforementioned concept of PM (Carver and Scheier, 1998; Ullsperger et al., 2014a). PM-related brain activity has repeatedly been linked to guiding the decision about whether and which action adaptation is warranted (Botvinick et al., 2001; Rushworth et al., 2004; Rushworth, 2008).

PM can be described as a set of continuously operating cognitive and affective functions that determine whether adaptive control is required and therefore provide the basis for successful goal-directed behavior (Ullsperger et al., 2014b). At a neural level, PM functions appear to be implemented by a PM network that comprises the anterior midcingulate cortex (aMCC), the pre-supplementary area (pre-SMA) and the adjacent dorsomedial prefrontal cortex (dmPFC) and is connected to the posterior medial frontal cortex (pMFC) (Rushworth, 2008; Nee et al., 2011; Shenhav et al., 2013; Ullsperger et al., 2014a). There is evidence, however, that the preSMA is more involved in inhibitory mechanisms and not conflict processing *per se* (Huster et al., 2011). Necessity, type and magnitude of adaptation are associated with signal changes in the pMFC (Ullsperger et al., 2014a). Various theories of PM exist, mainly differing with respect to the presumed information-processing mechanism generating the adaptation signal. While some theories assume that adaptation is employed based on a weighted prediction error signal (Holroyd and Coles, 2002; Alexander and Brown, 2011; Ullsperger et al., 2014b), others focus on the occurrence of information-processing conflicts and their detection by certain brain regions (mainly the aMCC), and propose that conflict signals serve as one aspect of a more general outcome monitoring function, which triggers strategic adjustment of cognitive control (Botvinick et al., 2004).

The ERN is a defined event-related potential (ERP) that is associated with PM at the response processing stage of goal-directed behavior (Falkenstein et al., 1991; Gehring et al., 1993; Ullsperger et al., 2014b). The ERN is an early frontocentral negativity which occurs on error trials peaking 50–100 ms after the response, and has been shown to be independent of stimulus and response effector modality (Falkenstein et al., 1991; Gehring et al., 1993; Ullsperger et al., 2014a). The ERN amplitude also appears to be influenced by subjective error significance (Endrass et al., 2007), and its source is assumed to be mainly localized within the anterior midcingulate cortex (aMCC) (Debener et al., 2005; Keil et al., 2010). The correct-related negativity (CRN) is a similar component that is observed following correct responses, but is reduced in amplitude (Ford, 1999; Endrass et al., 2012; Grützmann et al., 2014). During response processing, errors can be detected immediately when task rules are known (Danielmeier and Ullsperger, 2011). The ERN may therefore reflect fast alarm signals indicating the need to adapt behavior (Steinhauser and Yeung, 2010; Ullsperger et al., 2010; Wessel et al., 2011). The ERN amplitude has been shown to predict subsequent neural as well as within-task behavioral adjustments. This has been posited as evidence for the monitoring activity of the aMCC playing an important role in the employment of cognitive control or self-control (Kerns et al., 2004; Inzlicht and Gutsell, 2007). Evidence for this also arises from findings regarding altered PM correlates in mental disorders associated with deficient goal-directed control, such as OCD and SUD, which indicate a connection between neural measures of PM and regulatory control in daily life (Euser et al., 2013; Endrass and Ullsperger, 2014; Pasion and Barbosa, 2019). Stable individual differences in the ERN magnitude are considered a trait indicator for the disposition to recruit the control network and show adequate re-test reliability (Olvet and Hajcak, 2009; Fischer and Ullsperger, 2013; Riesel et al., 2013, 2014). Impaired self-control can be described as a deficient implementation of cognitive control—some models of self-control posit a balance between top-down control (as implemented by the prefrontal cortex) and subcortically mediated impulsive reactions to emotional stimuli or appetitive cues (Heatherton and Wagner, 2011; Hofmann et al., 2012b). These self-control failures (SCFs) can result from both underregulation and misregulation. Whereas, underregulation is a failure to exert self-control, misregulation entails the exertion of self-control, but in a misguided or counterproductive way (Baumeister and Heatherton, 1996). These connections are usually assessed *via* task performance or by using self-report questionnaires (de Ridder et al., 2012). However, there is data on links between PM and actual behavior outside the lab: In a study focused on emotion regulation, ERN difference scores also predicted the strength of the associations between daily stress and anxiety (Compton et al., 2008). Apart from error-related activity, higher inferior frontal gyrus (IFG) activity on correct trials of a go/no-go task has been connected to higher resistance to food-related temptations in daily life (Lopez et al., 2014). One study also more directly examined this association: Error-related activity inside the PM network in an fMRI study has been linked to self-control in daily life, as assessed by EMA. A higher amount of SCFs was associated with low error-related activation of the PM network involving aMCC, preSMA and anterior Insula, as well as low post-error IFG activation and less post-error slowing (Krönke et al., 2018).

The aim of the present study was to establish whether PM-related brain activity as indicated by the ERN predicts adaptive behavior in daily life, following the brain-as-predictor approach (Berkman and Falk, 2013). We specifically were interested in the employment of self-control in real-life situations involving conflicts between transient desires and superordinate or long-term goals. To this end, PM-related brain activity was assessed using electroencephalography (EEG) in an adapted version of the Eriksen flanker task (Eriksen and Eriksen, 1974). Self-control in daily life was measured utilizing EMA following procedures described by Wolff et al. (2016). We expected the ERN amplitude, as a measure of PM-related brain activity, to predict the amount of SCFs in daily life. Based on Krönke et al. (2018) we expected that lower amplitudes of the ERN, indicating attenuated error monitoring leading to a reduced behavioral adaptation and mobilization of cognitive control - should relate to higher propensity to commit SCFs in daily life.

## Methods

### Sample

One hundred and forty participants were recruited from the general population in the Dresden area. Seven participants made more than 40% errors across all trials, one had a significant number of random button presses, and another had discontinued the assessment. They were therefore excluded from further analyses. The final sample consisted of 131 participants (58.8% female; *M* = 25.86 years, *SD* = 5.65), 121 participants (92.4%) had completed advanced education degrees, 9% reported past mental health problems. 95.4% of participants self-identified as of mainly European, 4.6% as of mainly Asian ancestry. All participants had normal or corrected-to-normal vision, were native speakers of German, and reported no history of head trauma or neurological disease. Participants were further not included if they reported taking psychotropic substances within the past 3 months; reported a history of bipolar disorder, borderline personality disorder, psychotic episodes, or severe alcohol use disorder; currently met the criteria for an eating disorder or severe episode of major depression; reported a lifetime use of illicit substances of more than twice a year and lifetime use of cannabis of more than twice a month.

The study was conducted in accordance with the ethical guidelines of the Declaration of Helsinki. The ethics committee at the University Hospital Carl Gustav Carus, Technische Universität Dresden approved study procedures (EK 372092017). All participants gave informed consent.

### Procedure, Measures, and Tasks

#### Procedure

Participants completed two sessions in the lab, and between those sessions a week of EMA of self-control in daily life. Questionnaire data were obtained during the first session, at the end of which participants received a smartphone for EMA and completed a short EMA tutorial. The tutorial comprised answering the EMA questionnaire regarding five fictive situations and participants could ask questions and received feedback. PM-related brain activity was assessed using the ERN during a flanker task using EEG during the second session. We also assessed correct-related activity in incongruent trials, as well as the difference between error- and correct-related activity in incongruent trials, to distinguish between error-sensitive and outcome-independent aspects of response monitoring (Endrass et al., 2012; Grützmann et al., 2014). The EEG session took place at least 8 days after the first session. During both sessions, participants completed other tasks, which are not part of this report.

#### Ecological Momentary Assessment

We assessed self-control in daily life using EMA during a seven-day period similar to Wolff et al. (2016) and Hofmann et al. (2012a), assessing the occurrence of desires, how strong they were, if these desires were conflict-laden, how strong the conflict was, if participants tried to resist the desire and if they enacted the desire. Up to four dichotomous variables (desire, conflict, resistance, and enactment), one categorical variable (desire type), and two continuous variables (desire and conflict strength) were acquired per questionnaire. Participants were instructed to carry the devices with them at all times during the assessment window. Depending on response rates, participants completed up to 56 questionnaires. SCFs were operationalized as enactments of conflict-laden desires divided by the number of questionnaires participants had responded to. Self-control is required when one experiences a desire that conflicts with a long-term goal or a personal standard (Hofmann et al., 2012a). Participants received eight short questionnaires throughout the day, delivered within a 14 h time window, which was chosen based on participant's usual waking hours (starting at either 8, 9, or 10 a.m.). The time points were randomized, but at least 1 h apart and signaled by an alarm. Alarms could be manually deferred by participants, for a maximum of 15 min. Participants received identical smartphones (Nokia 5). A customizable EMA application delivered the questionnaires, all other functions were blocked (movisensXS, version 1.3.3; movisens GmbH, Karlsruhe, Germany). See Supplementary Figure 2 for a schematic depiction of a questionnaire. Internal consistency of SCFs was acceptable, determined as the Spearman-Brown corrected split-half reliability, using the odd-even method (0.77).

#### Flanker Task

**Figure 1:**
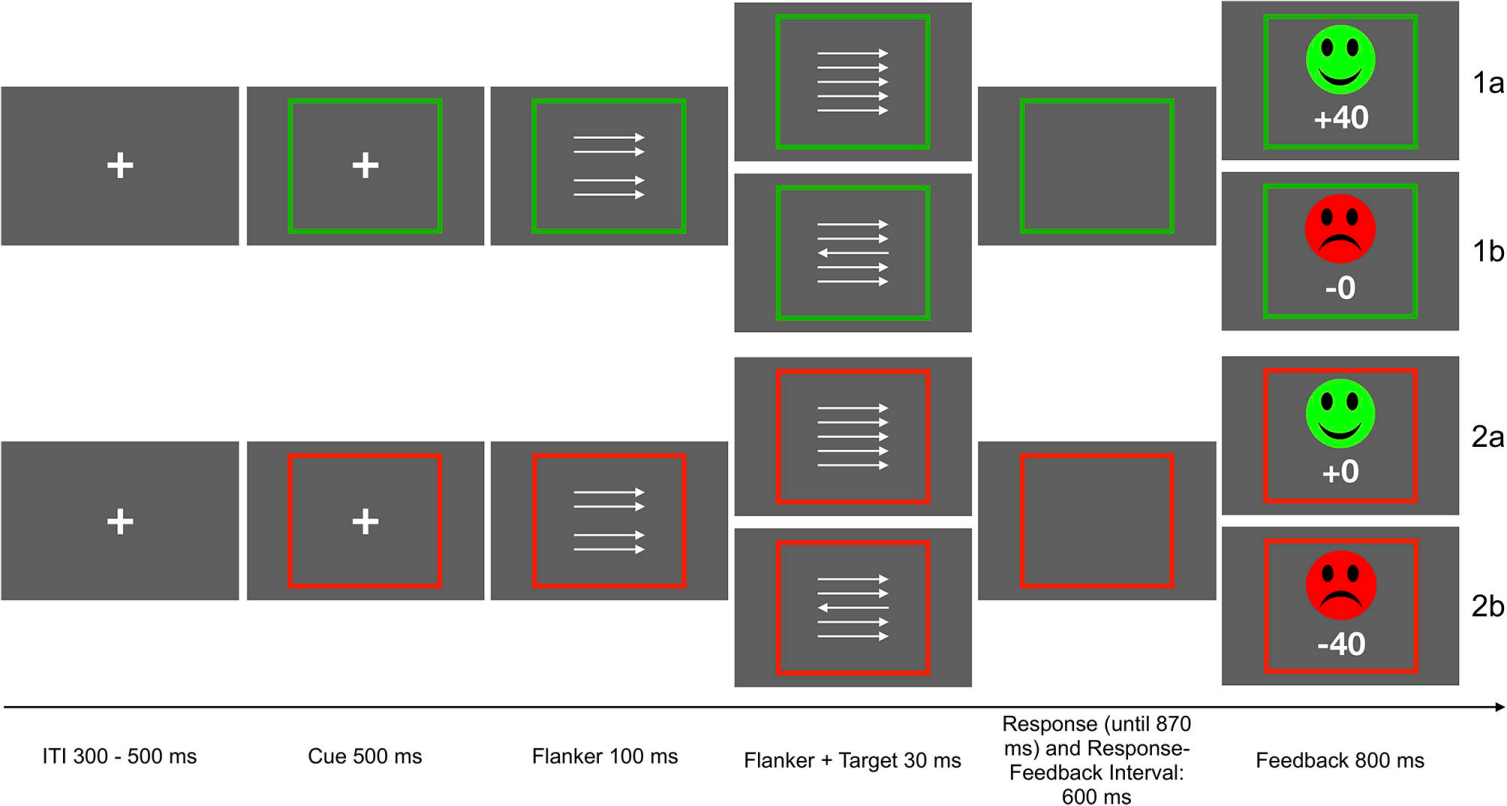
Schematic depiction of the flanker task. Participants were instructed to respond with the left or right button according to the direction of the middle arrow. In the gain context, the fastest 20% of the correct response were rewarded (1a), in the loss avoidance context incorrect and slowest responses were punished (2b). The other responses were neither punished nor rewarded (1b, 2a).

Participants performed a modified version of the arrow-version of the Eriksen flanker task (Eriksen and Eriksen, 1974; Kopp et al., 1996). The flanker stimuli consisted of four vertically arranged arrows pointing to the left or the right. A fifth arrow appeared as target stimulus with a delay of 100 ms, in addition to the surrounding flanker arrows. All arrows remained on screen for 30 ms. In 50% of the trials, the target stimulus pointed in the same direction (congruent) as the surrounding arrows. In the other 50% of the trials, the target pointed in the opposite direction (incongruent). Participants had to respond using a left or right button, according to the direction of the target arrow. The task was presented in two incentive contexts which were cued as follows: Each trial started with an incentive cue, a green or red frame surrounding a fixation cross, signaling potential gain (green) or loss (red) of 40 points in the current trial (presented for 500 ms). The frame remained visible for the duration of the trial. In the gain condition (50% of all trials), the fastest 20% of the correct responses were rewarded (40 points) while errors resulted in reward omission (0 points). In the loss avoidance condition, incorrect and the slowest responses were punished (minus 40 points), and correct responses resulted in punishment omission. Slowest responses were defined by an adaptive deadline based on individual performance and response time, in order to obtain a rate of 20 % negative feedback for each context. Performance feedback was presented for 800 ms after a response interval of 900 ms following target onset or 600 ms after response. The deadline was initially set at 500 ms, and was adapted based on the rate of negative feedback. If the rate of negative feedback was higher than 20%, 30 ms were added to the adaptive deadline, if the rate was below 20%, 30 ms were subtracted from the deadline. The two incentive contexts were introduced to the task for a different research question than the one addressed here. However, associations with SCF were examined separately for the two incentive contexts, and reported as supplementary results. Associations were significant in both contexts and exhibited similar effect sizes. Participants could earn a bonus of up to 5 EUR, depending on task performance and points earned. They received 4 EUR if they earned less than 5,000 points, 4,50 EUR for 5,001 to 5,500 points, and 5 EUR for more than 5,501 points. The task included 640 trials of 2.53 to 2.75 s duration. The task was presented using Presentation 19.0 (Neurobehavioral Systems Inc., Berkeley, CA, USA). See Figure 1 for a schematic depiction of the task.

### Psychophysiological Recording and Data Reduction

The EEG was continuously recorded at a sampling rate of 500 Hz using elastic EEG caps with 63 Ag/AgCl electrodes at equidistant locations (EasyCap GmbH, Herrsching-Breitbrunn, Germany) and two 32-channel BrainAmp amplifiers (Brain Products GmbH, Munich, Germany). Impedances were kept below 10 kOhm. Two external electrodes placed below the left and right eye were used to capture eye movement. Ground and reference electrodes were placed next to Fz (at AFF1h and AFF2h, theta/phi spherical coordinates:−58/78 and 58/78). Offline analyses were performed using EEGLAB 14.1.2 (Delorme and Makeig, 2004) and MATLAB 2018b (The MathWorks Inc., 2018). The EEG was high- and low-pass filtered with cutoffs of 0.1 and 30 Hz, respectively, and epoched from −500 to 2,000 ms relative to target stimulus onset. Epochs with artifacts were rejected automatically based on signal deviations >5 SD of the mean probability distribution on any single channel or the whole montage. Remaining epochs were demeaned and submitted to adaptive mixture independent component analysis (AMICA) implemented in EEGLAB. Independent components reflecting ocular or cardiovascular artifacts were removed manually and EEG data were re-referenced to common average reference. Subsequently, response-locked epochs from −500 to 1,000 ms were created. The average EEG activity 400 to 200 ms prior to response was used as baseline. Individual participant's mean amplitudes per trial type were calculated for each time point and electrode within the extracted epochs. Internal consistency of EEG measures was excellent, determined as the Spearman-Brown corrected split-half reliability (odd-even method) for error and correct amplitudes (0.92, 0.98), respectively, averaged for FCz, Fz, F1, and F2 in a time window of 20 ms around the individual peak.

### Data Analysis

An adaptive elastic net regularized (aenet) regression based on the Poisson distribution was fit to predict SCFs from response-locked EEG (Zou and Hastie, 2005; Zou and Zhang, 2009; James et al., 2013). The model was run using within-subject averaged response-locked EEG activity of incongruent error trials. Two additional models were run: one using difference scores of the within-subject averaged response-locked EEG for incongruent error minus incongruent correct trials and one using the within-subject averaged response-locked EEG for incongruent correct trials. The aenet regression selects the variables relevant for the prediction of an outcome from an ultra-high dimensional dataset, a dataset in which the number of predictors outnumber the amount of observations, by setting the coefficients of non-relevant features to zero (Zou and Hastie, 2005; Zou and Zhang, 2009). However, it also allows the grouped selection of correlated features, which is highly relevant when dealing with EEG data (Zou and Hastie, 2005; Zou and Zhang, 2009). We also chose the aenet regression because in the analysis of ultra-high dimensional data, a method should have oracle properties in addition to yielding sparse models, meaning that it consistently identifies the right subset model and has an optimal estimation rate (Fan and Li, 2001; Fan and Peng, 2004; Zou, 2006; Zou and Zhang, 2009). The predictors, or features, in this case were the EEG signals at each electrode and time point. Epochs were selected so that they would contain relevant time points for response-processing but also reduce the amount of features, so that estimation would be more reliable (James et al., 2013). We chose an epoch from 50 ms prior to the response to 300 ms after the response. A central cluster of 23 electrodes was also chosen for relevance in response processing (including Cz, FCz, FC2, CP2, CPz, CP1, FC1, Fz, F2, FC4, C4, CP4, P2, Pz, P1, CP3, C3, FC3, F1, PO4, PO2, PO1, PO3). This resulted in 4,025 data points (23 electrodes × 175 time points). In order to be able to compute accurate estimates within a relatively small sample, we tuned the alpha and lambda hyperparameters using 5-fold cross validation with 10 repeats within an elastic net regression. We used the root-mean-square error (RMSE) as the metric for optimization. Then, the adaptive penalty factors were estimated using the estimated beta values from the first model with the best tuning results. Using these adaptive penalty factors, the aenet regression was tuned using 5-fold cross validation with 10 repeats. Finally, an aenet regression using the final aenet model hyperparameters was internally validated using 0.632 bootstrapping (Efron, 1983), with 1,000 bootstrap samples. We chose bootstrapping for internal validation over external cross validation using training and test data, because in smaller datasets this has been shown to reduce bias and improve model performance (Harrell, 2015). We then estimated the importance of predictors in the model using the absolute values of the coefficients corresponding to the tuned model. Variable importance represents the effect of an predictor on the output of a model when inputs are varied (Kuhn et al., 2020).

In addition, generalized linear models (GLMs) based on negative binomial distribution were built to predict SCFs from mean ERN amplitude in a time window of 20 ms around the individual ERN peak, averaged for FCz, Fz, F1, and F2, as well as from behavioral measures. See the supplement for GLMs predicting SCFs from difference scores and the mean CRN amplitude, also based on a time window around the individual peak. Because the behavioral data were not normally distributed, we analyzed differences between behavioral measures using a bootstrapped version of Yuen's test. The difference between incongruent error trials and incongruent correct trials was tested for each time point within an epoch from 50 ms prior to the response to 300 ms after the response in electrodes Fz and FCz, using dependent sample *t*-tests with Bonferroni-corrected alpha. Statistical analysis of EEG and behavioral data was performed in R 4.0.0 (R Core Team, 2020) using the packages glmnet v4.0, msaenet v3.1, caret v6.0-86, MASS v7.3-51.5, and WRS2 v1.0-0 (Venables and Ripley, 2002; Kuhn, 2008; Friedman et al., 2010; Xiao and Xu, 2015; Mair and Wilcox, 2019) and MATLAB 2018b (The MathWorks Inc., 2018).

## Results

### Behavioral Results

#### Ecological Momentary Assessment

On average, participants responded to 48.52 (*SD* = 7.59, *MD* = 51) of the 56 issued alarms (86.64 %). In 70.19% of answered alarms participants reported desires, 38.22% of desires were conflict-laden, and of those 56.60% were enacted. See Table 1 and Supplementary Figure 1 for further results.

**Table 1:** Measures (means, medians, and standard deviations) in ecological momentary assessment.

	**M**	**MD**	**SD**
Desires	34.05	36	12.20
Desire strength	2.72	2.82	1.05
Conflict	13.02	12	8.64
Conflict strength	0.91	0.88	0.61
Resistance	8.98	8	6.63
Enactment	25.66	27	10.15
SCFs	0.15	0.13	0.12

*Desires, number of experienced desires; Desire strength, strength of experienced desires on scale of 1-6 (low to high); Conflict, number of conflicted desires; Conflict strength, strength of experienced conflict on scale of 1-6 (low to high); Resistance, number of desires that participants tried to resist; Enactment, number of enacted desires; SCFs, self-control failures, number of conflict-laden desires that were enacted divided by answered questionnaires*.

#### Flanker Task

Average error rate was 16.75% (*SD* = 8.22%). The propensity for committing an error was significantly reduced on post-error trials compared to post-correct trials, *Y*_*t*_ = 0.04 (95% CI: 0.02 0.05), *p* < 0.001, providing evidence of improved accuracy resulting from post-error adaptations. Reaction time (RT) was significantly higher on correct than error trials, *Y*_*t*_ = 67.50 (95% CI: 65.37 69.63), *p* < 0.001. For correct trials, RT was significantly higher on incongruent than congruent trials, *Y*_*t*_ = 89.41 (95% CI: 85.78 93.04), *p* < 0.001, providing evidence for an interference effect (Cohen et al., 2000). For further analyses, trials were categorized into pre-error (correct trials before error commission), error, post-error (correct trials after error commission), and post-correct trials (correct trials following other correct trials) (Danielmeier et al., 2011). These trials also differed in RT: Error RT were significantly faster than post-error RT, *Y*_*t*_ = 72.05 (95% CI: 68.89 75.20), *p* < 0.001, post-correct RT, *Y*_*t*_ = 65.50 (95% CI: 63.25 67.74), *p* < 0.001, and pre-error RT, *Y*_*t*_ = 49.42 (95% CI: 47.05 51.78), *p* < 0.001. Post-error RT were significantly slower than post-correct RT, *Y*_*t*_ = 6.55 (95% CI: 4.17 8.93), *p* < 0.001. Pre-error RT were significantly faster than both post-error and post-correct RT, respectively, *Y*_*t*_ = 22.63 (95% CI: 25.20 −20.06), *p* < 0.001, and *Y*_*t*_ = 16.08 (95% CI: 18.16 13.99), *p* < 0.001. Trials in the loss avoidance condition had slightly longer RT, compared to the gain condition (all *p* < 0.05), but this was not the case on incongruent correct trials. We did not find evidence of post-error slowing, when comparing RT of post-error and post-correct incongruent trials, *Y*_*t*_ = 0.07, *p* = 0.93. Behavioral results are also presented in Table 2.

**Table 2:** Task performance and measures of performance monitoring (means and standard deviations) in the flanker task.

	**M**	**SD**
**Correct Trials**		
Correct incongruent RT in ms	380.20	32.95
Correct congruent RT in ms	290.33	32.60
**Trials Around Errors**		
Error RT in ms	259.52	29.43
Post-correct RT in ms	324.13	35.16
Post-error RT in ms	330.75	39.23
Pre-error RT in ms	307.95	33.40
**Accuracy**		
PEA in %	77.45	15.37
PCA in %	73.86	14.42

*RT, reaction time; Error reaction times refer to all error trials; Pre-error reaction times refer to correct trials before error commission; Post-error reaction times refer to correct trials after error commission; Post-correct reaction times refer to correct trials after correct responses; PEA, Post Error Accuracy; PCA, Post Correct Accuracy*.

#### Associations Between Behavioral Measures and SCFs

There was no significant association between SCFs and post-error slowing (*p* = 0.61), operationalized as the RT difference between post-error and post-correct incongruent trials, error rate (*p* = 0.15), post-error accuracy (*p* = 0.17), post-correct accuracy (*p* = 0.17), and the interference effect (*p* = 0.64), operationalized as the RT difference between incongruent and congruent correct trials. Internal consistency of PES and the interference effect (0.67, 0.94), respectively, was determined as the Spearman-Brown corrected split-half reliability, using the odd-even method.

### EEG Analysis

The ERN peaked at 48 ms at electrode FCz, and at 46 ms at Fz (local minimum of grand average). Peak electrode was FCz. The difference between error trials and correct trials following the response was significant between 0 and 104 ms at FCz (all *p* < 0.0001), and 28 and 110 ms for Fz (all *p* < 0.0001).

**Figure 2:**
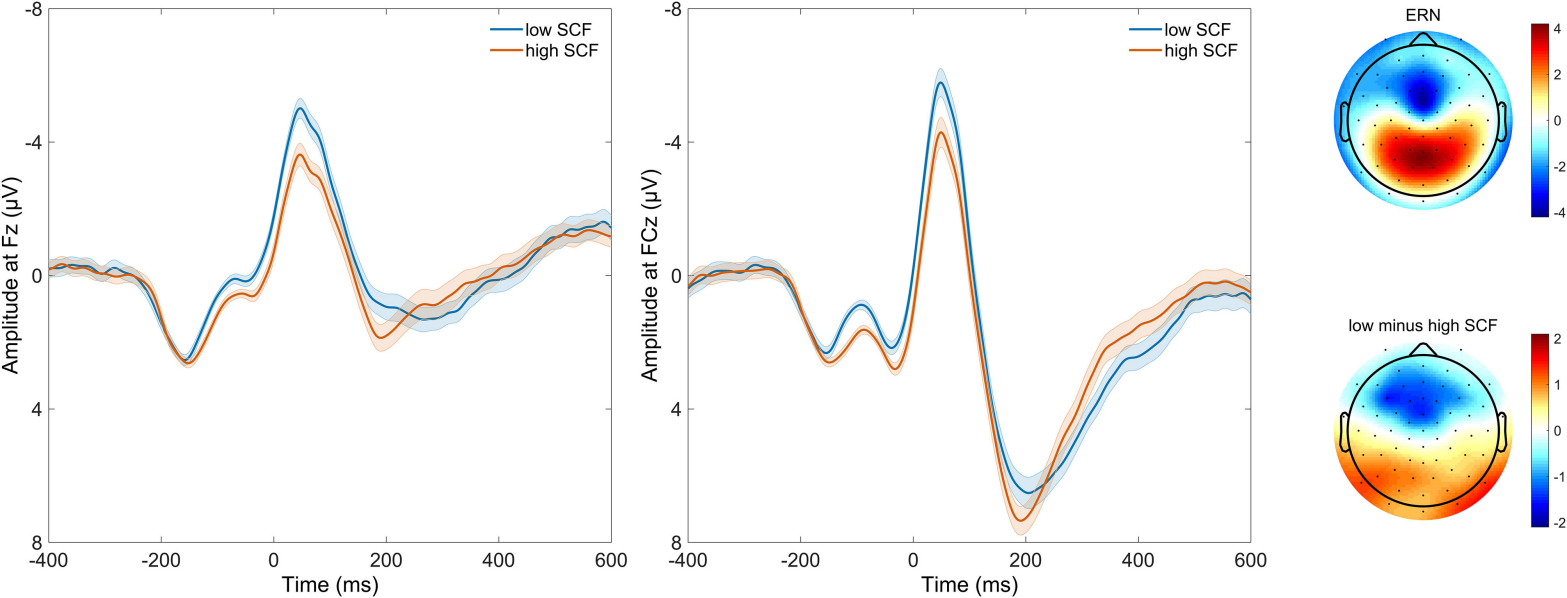
Time course of response-locked EEG activity at electrodes FCz and Fz, and scalp distribution of response-locked EEG activity as well as difference in EEG activity between high and low number of self-control failures. Grand average event-related potential (ERP) waveforms are depicted on the left, response-locked for incongruent error trials. Waveforms are, for purpose of visualization, split by median and plotted separately for those with a lower (loSCF, blue) and those with a higher amount of self-control failures (hiSCF, red). Shadows indicate the SEM. Scalp distribution of response-locked EEG activity for ERN effect at 64 ms, as well as the difference in EEG activity at 64 ms between those with a lower and those with a higher amount of self-control failures are depicted on the right, as split by median for visualization.

**Figure 3:**
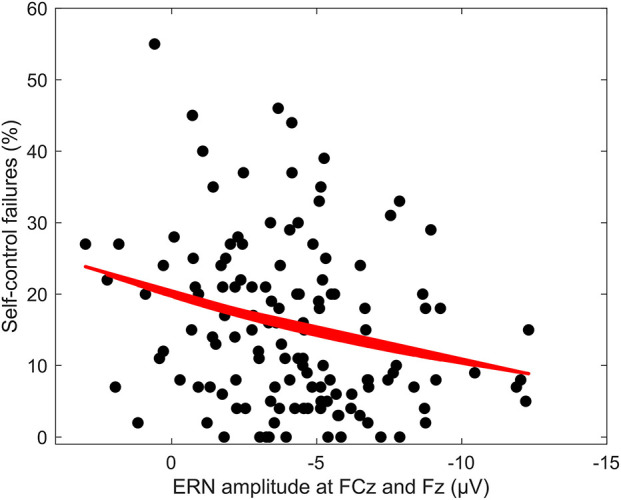
Regression of ERN amplitude at FCz and Fz on SCF at 60 to 70 ms. Regression of mean ERN amplitude at FCz and Fz between 60 and 70 ms response-locked for incongruent error trials on amount of self-control failures per answered questionnaire.

Averaged response-locked EEG epochs for incongruent error trials were submitted to aenet regression analysis with SCFs as outcome; EEG signals at each electrode and time point served as predictors [final model: α = 0.75, λ = 0.0273, RMSE = 2.05, *R*^2^ = 0.04, mean absolute error (MAE) = 2.04]. This analysis revealed four variables of importance with predictive value for SCFs (see Table 3). All of these coefficients corresponded to fronto-central electrode Fz and time points associated with the ERN in the averaged event-related potentials. Therefore, the ERN amplitude of error trials in a flanker task significantly predicted the amount of SCFs at a frontal electrode site. Higher, or more negative, ERN amplitudes predicted less SCFs and mean amplitude of the ERN was reduced in individuals reporting a higher number of SCFs. See Figure 2 for a visualization of ERP waveform and scalp distribution for the ERN and Figure 3 for visualization of the association. Additionally, difference scores of the averaged response-locked EEG epochs for incongruent error minus incongruent correct trials were also submitted to aenet regression analysis with SCFs as outcome (final model: α = 0.75, λ = 0.0213, RMSE = 2.05, *R*^2^ = 0.03, MAE = 2.04). Variables of importance corresponded to fronto-central electrodes and time-points associated with the ERN (see Table 3). Lastly, averaged response-locked EEG epochs for incongruent correct trials were submitted to aenet regression analysis with SCFs as outcome (final model: α = 0.75, λ = 0.0208, RMSE = 2.05, *R*^2^ = 0.03, MAE = 2.04). All variables of importance corresponded to posterior-occipital electrode PO1 and time-points prior to the response (see Table 3). Therefore, we found no evidence that the CRN amplitude, unlike the ERN, predicted the amount of SCFs and the effect was specific for error-related activity.

**Table 3:** Most important variables with predictive value for SCFs defined by electrode and timepoint.

	**Electrode**	**Time point (ms)**	**Estimate**
Incongruent error trials	Fz	62	0.001410
	Fz	64	0.008173
	Fz	66	0.008735
	Fz	68	0.002214
Incongruent correct trials	PO1	−38	−0.003124
	PO1	−36	−0.007063
	PO1	−34	−0.004974
	PO1	−32	−0.000045
Difference scores	Fz	54	0.000001
	Fz	56	0.000502
	FC3	56	0.004293
	FC3	58	0.009801
	FC3	60	0.002800

*SCFs, self-control failures; time point, time point within response-locked epoch including 50 ms before and 300 ms after response; estimate, estimate of regression weight for the respective predictors*.

Individual peak ERN amplitude at electrodes FCz, Fz, F1, and F2 also predicted SCFs within a GLM based on negative binomial distribution (β = 0.058, *z* = 2.097, *p* < 0.05).

## Discussion

The present study investigated the association between neural correlates of PM in a flanker task and self-control in daily life as measured by EMA. Results showed that higher ERN amplitudes predicted fewer SCFs, indicating higher self-control in daily life. Results therefore support an association between lab-based assessment of neural correlates of PM and self-control in daily life, as our analysis revealed four variables of importance corresponding to the ERN time window in an analysis that included 23 electrodes and all time points from 50 ms before until 300 after the response. In addition, this association was also shown by a regression with the mean amplitude for the ERN at frontocentral electrodes in a time window around the individual peak. The association was also observed for the difference between error- and correct-related activity, but no relationship between SCFs and CRN was observed. As adaptive, goal-directed behavior depends on monitoring for the need to adapt behavior, and ERN amplitudes have been interpreted as reflecting the detection of errors or unexpected action outcomes, indicating the need to adapt (Steinhauser and Yeung, 2010; Ullsperger et al., 2010; Wessel et al., 2011), a connection between PM and self-control seems plausible. This connection has been established before, using fMRI (Krönke et al., 2018). However, despite PM being necessary for appropriate recruitment of control, it is not sufficient, as additional brain regions have to respond to these signals for adaptive control to be implemented (Botvinick et al., 2001). This might also account for the small size of the effect within our sample.

Overall, our results fit well into the literature on self-regulation and self-control (Inzlicht et al., 2020). PM appears to exhibit parallels to the monitoring function described in the self-regulation literature and predicts self-control in daily life and therefore the implementation of actions according to desired goals (Baumeister and Heatherton, 1996; Carver and Scheier, 1998). Hofmann and Kotabe (2012) proposed a taxonomy of different SCFs, including self-monitoring failures, motivational SCFs and volitional SCFs, and distinguish between preventive (anticipatory) and interventive (momentary) self-control. Whereas interventive self-control refers to inhibition of immediate impulses, preventive self-control includes initiating desired, goal-directed behavior as well as preventing encounters with situations, which may elicit temptations (Myrseth and Fishbach, 2009; de Ridder et al., 2011). A related concept from cognitive neuroscience is the distinction between preparatory and corrective control (Cohen et al., 2000) or the distinction between proactive and reactive control (Braver, 2012). SCFs as assessed in this study probably fall in the categories of motivational and volitional SCFs, as the preceding desire and conflict are reported and self-monitoring was therefore successful. An influence of motivation on how successfully individuals exert self-control has frequently been reported (Muraven and Slessareva, 2003; Hofmann and Kotabe, 2012; Vohs et al., 2012). Having low motivation for self-control might result in fewer reported conflict-laden desires, and thus, SCFs. At the same time, individuals with higher motivation might report more conflict-laden desires, while at the same time also trying to avoid situations in which they might encounter a desire. However, individuals high in self-control have been reported to experience desires less frequently (Bernecker et al., 2018). This could indicate that individuals higher in self-control may avoid situations in which they might be tempted because of a high subjective significance of not failing at the implementation of self-set goals, and subsequently experience less desires. This would also fit well with the reported modulations of the ERN amplitude by subjective error significance (Endrass et al., 2010; Wessel et al., 2012): Individuals high in self-control may attach great importance to sticking to their goals and performing well, which is consequently reflected in higher error-related brain activity as indicated by the ERN.

Higher ERN amplitudes predicting less SCFs therefore presents as an intuitive connection at first. Individuals with higher ERN amplitudes appear to possess a more effective monitoring system to signal a higher need for the employment of cognitive control when needed, while at the same time attaching more significance to errors, resulting in better self-control (Inzlicht and Gutsell, 2007; Endrass et al., 2010). The ERN has been shown to have high re-test reliability, and changes in ERN amplitude have been observed in psychopathology (Riesel et al., 2013; Pasion and Barbosa, 2019). But when thinking about individuals with OCD, who display impaired self-control in daily life despite consistent evidence on higher ERN amplitudes (Endrass and Ullsperger, 2014), questions about how our findings relate to enhanced ERN in individuals with OCD do arise. Larger ERN amplitudes in OCD appear to be unrelated to performance or adjustment of behavior within-task, such as post-error slowing. This could suggest deficient translation of monitoring signals into adaptive behavior and therefore a failure to implement control (Endrass et al., 2010; Jansen and de Bruijn, 2020). Another explanation, which might fit with clinical representations of OCD, might be misguided self-control: such that in OCD self-control is applied in an inadequate or counterproductive manner (such as washing hands 50 times a day to prevent infection transforms a usually adaptive behavior—hand washing—into a self-control problem, presenting as excessive behavior) (Heatherton and Wagner, 2011). The concept of self-control could therefore also be discussed in terms of how much and what kind of self-control is “healthy,” and what kind of behavior is excessive, misguided and costly (Goschke, 2014). Other disorders, more on the externalizing spectrum, like SUD or pathological gambling, might fit more with the framework of underregulation, as those disorders appear to reflect a failure to exert self-control when needed (Luijten et al., 2014). Consistent with our present findings, externalizing disorders (including a general aggressive disposition) have been associated with lower ERN amplitudes (Troller-Renfree et al., 2018; Grisetto et al., 2019; Pasion and Barbosa, 2019).

Our finding that a reduced ERN predicts a higher frequency of SCFs is fully consistent with Krönke et al. (2018) result that reduced activation the PM network as assessed with fMRI predicts a higher propensity to commit SCFs. However, results are less consistent with respect to error rates and interference effects. While Krönke et al. (2018) did find evidence for an association between error rates, post-error slowing, and SCFs, this was not the case in our study. One possible explanation for this discrepancy may reside in the different tasks used in our and the Krönke et al. (2018) study (flanker versus counting Stroop, respectively). Within-task adjustments of behavior, reflecting enhanced goal-directed control, have been associated with higher aMCC activity on high-conflict and error trials (Kerns et al., 2004; Danielmeier and Ullsperger, 2011; Danielmeier et al., 2011). Implementation of these behavioral adjustments, however, seems to be mediated by lateral PFC regions, and aMCC engagement has been proposed to reflect signaling for an enhanced recruitment of top-down control (Kerns et al., 2004). However, whether behavioral adaptation effects in such tasks actually reflect control adjustments or whether they can be accounted for by non-control related processes has been questioned (Braem et al., 2019; Schmidt, 2019). Consequently, there have been demands for analysis of other measures of control implementation, such as ecologically more valid assessment of self-control *via* EMA (Notebaert et al., 2009; Danielmeier et al., 2011; Gehring et al., 2018; Meyer and Hajcak, 2019). Also, the current version of the flanker task including feedback shortly after every response, even punishing correct responses when they were too slow and thereby giving false external feedback, might hinder the emergence of post-error slowing (Saunders and Jentzsch, 2012). In addition, the reliability (0.67) of our post-errors slowing measure (as RT difference) was insufficient [see also Hedge et al. (2018)]. It therefore remains a question for future research, whether the connection between neural correlates of PM and control recruitment may be more valid predictors of self-control in daily life than behavioral adjustments in laboratory tasks. In addition, current findings should be replicated using a standard flanker task (without task contexts and feedbacks). However, irrespective of these unresolved issues, the present results add to a growing body of evidence documenting associations between neural indicators of monitoring and control processes with ecologically valid assessments of real-life self-control *via* EMA.

How neural correlates of PM relate to regulatory control in daily life is central to our understanding of the complex mechanisms underlying goal-directed behavior. Other aspects of the connection uncovered in the current study have to be examined in future studies. SCFs reported by participants of the present study most likely represent instances, in which interventive self-control would be required to resist current temptations and to support the pursuit of long-term goals. However, the number of SCFs might also partly reflect individual differences in the use of preventive self-control strategies (e.g., pre-commitment), which might help to avoid temptations and self-control conflicts and thus result in fewer reported desires and conflicts. Whether PM differentially influences interventive and preventive self-control remains an interesting but unresolved question (de Ridder et al., 2011; Hofmann and Kotabe, 2012). As mentioned above, it would also be of interest to examine the connection between PM and the actual neural implementation of self-control and how that in turn relates to goal-directed behavior in daily life. It has been suggested that the exertion of self-control is implemented *via* the top-down-modulation of task-relevant perceptual representations in conflict tasks (e.g., the Stroop task) (Kerns et al., 2004; Krönke et al., 2018) and of value representations in choice conflict tasks (Hare et al., 2009; Krönke et al., 2020) by goal representations maintained in the dorsolateral prefrontal cortex. Moreover, such top-down modulations have been shown to predict real-life self-control. Thus, bridging the gap between PM and the implementation of self-control in laboratory tasks, and assessments of goal-directed behavior in daily life will be particularly important for understanding mechanisms underlying deficient or misguided self-control in various mental disorders. Moreover, it could also help to understand how to effectively implement public policies aiming at a reduction of harmful behavior and SCFs (Duckworth et al., 2018). Of particular interest is the role of affect in conflict monitoring, which has been addressed within the framework of the affective-signaling hypothesis (Dignath et al., 2020). Another focus could be the specificity of the influence of PM on different kinds of SCFs, for example comparing dietary SCFs with SCFs in social situations. To summarize, research aiming to elucidate more aspects of self-control in daily life as well as studies connecting PM and self-control in daily life to within-task measures of neural implementation of control are warranted.

Limitations of the current study include that even though EMA has been shown to have high ecological validity, compared to other methods, as it does measure behavior in daily life, it cannot be excluded that the assessment itself has effects on behavior. Because participants are instructed to monitor themselves, EMA could function as an intervention and thus dampen ecological validity (Ram et al., 2017). Regarding the analysis, while aenet regression does improve prediction accuracy and robustness by shrinking estimated parameters or setting them to zero and can handle correlated predictors, it does not take into account the structure of the data that is spatially and temporally correlated (Grosenick et al., 2013; Engebretsen and Bohlin, 2019). Depending on the tuned hyperparameters it is also possible that the aenet selects just one subgroup of correlated variables as a representative for the correlated predictors. Consequently, important variables might be missed in the presence of this subgroup of variables correlated with them (Grömping, 2009). This may also be an explanation for why ERN time points at other frontocentral sites failed to be selected as predictors. Future studies should consider these aspects, increasing interpretability. Aside from these measurement and analysis concerns, our sample size could have been larger to optimize prediction.

The ERN, as a neural correlate of PM processes, appears to predict self-control in daily life. Previously, regulatory control has mainly been assessed using within-task performance measures. However, such measures have been criticized for their lack of ecological validity. Our design sought to remedy these shortcomings, and we were able to establish a more ecologically valid connection between lab-based assessment of PM and self-control in daily life. Altered cognitive control processes and PM have been proposed as underlying mechanisms for various mental disorders (Goschke, 2014), and the ERN amplitude in particular has been shown to be associated with various psychopathologies, including OCD and SUD (Weinberg et al., 2015). Understanding how alterations in PM relate to regulatory control might therefore aid in delineating the type of deficit exhibited and developing targeted treatment strategies for affected individuals.

## Data Availability Statement

The original contributions presented in the study are included in the article/Supplementary Material, further inquiries can be directed to the corresponding author/s.

## Ethics Statement

The studies involving human participants were reviewed and approved by the ethics committee at the University Hospital Carl Gustav Carus, Technische Universität Dresden. The patients/participants provided their written informed consent to participate in this study.

## Author Contributions

RO conducted the data analysis and wrote the manuscript text. TE and TG designed the study. RO and JB organized data collection. TG and MW provided support and methodology for the EMA assessment. TE and RD supervised data analysis. All authors contributed to interpretation and reviewed the manuscript.

## Conflict of Interest

The authors declare that the research was conducted in the absence of any commercial or financial relationships that could be construed as a potential conflict of interest.

## References

[B1] Alexander W. H. Brown J. W. (2011). Medial prefrontal cortex as an action-outcome predictor. Nat. Neurosci. 14:1338. 10.1038/nn.2921 21926982 PMC3183374

[B2] Baumeister R. F. (2014). Self-regulation, ego depletion, and inhibition. Neuropsychologia 65, 313–319. 10.1016/j.neuropsychologia.2014.08.012 25149821

[B3] Baumeister R. F. Heatherton T. F. (1996). Self-regulation failure: an overview. Psychol. Inquiry 7, 1–15. 10.1207/s15327965pli0701_1

[B4] Berkman E. T. Falk E. B. (2013). Beyond brain mapping: using neural measures to predict real-world outcomes. Curr. Direct. Psychol. Sci. 22, 45–50. 10.1177/0963721412469394 24478540 PMC3903296

[B5] Bernecker K. Job V. Hofmann W. (2018). Experience, resistance, and enactment of desires: differential relationships with trait measures predicting self-control. J. Res. Personal. 76, 92–101. 10.1016/j.jrp.2018.07.007

[B6] Botvinick M. M. Braver T. S. Barch D. M. Carter C. S. Cohen J. D. (2001). Conflict monitoring and cognitive control. Psychol. Rev. 108:624. 10.1037/0033-295X.108.3.624 11488380

[B7] Botvinick M. M. Cohen J. D. Carter C. S. (2004). Conflict monitoring and anterior cingulate cortex: an update. Trends Cogn. Sci. 8, 539–546. 10.1016/j.tics.2004.10.003 15556023

[B8] Braem S. Bugg J. M. Schmidt J. R. Crump M. J. Weissman D. H. Notebaert W.. (2019). Measuring adaptive control in conflict tasks. Trends Cogn. Sci. 23, 769–783. 10.1016/j.tics.2019.07.002 31331794 PMC6699878

[B9] Braver T. S. (2012). The variable nature of cognitive control: a dual mechanisms framework. Trends Cogn. Sci. 16, 106–113. 10.1016/j.tics.2011.12.010 22245618 PMC3289517

[B10] Carver C. Scheier M. (1998). On the Self-Regulation of Behavior. New York, NY: Cambridge 10.1017/CBO9781139174794

[B11] Cohen J. D. (2017). “Cognitive control: core constructs and current considerations,” in Wiley Handbook of Cognitive Control, 1–28. Available online at: https://psycnet.apa.org/record/2017-19774-001. 10.1002/9781118920497.ch1

[B12] Cohen J. D. Botvinick M. Carter C. S. (2000). Anterior cingulate and prefrontal cortex: who's in control? Nat. Neurosci. 3:421. 10.1038/74783 10769376

[B13] Compton R. J. Robinson M. D. Ode S. Quandt L. C. Fineman S. L. Carp J. (2008). Error-monitoring ability predicts daily stress regulation. Psychol. Sci. 19, 702–708. 10.1111/j.1467-9280.2008.02145.x 18727786

[B14] Danielmeier C. Eichele T. Forstmann B. U. Tittgemeyer M. Ullsperger M. (2011). Posterior medial frontal cortex activity predicts post-error adaptations in task-related visual and motor areas. J. Neurosci. 31, 1780–1789. 10.1523/JNEUROSCI.4299-10.2011 21289188 PMC6623722

[B15] Danielmeier C. Ullsperger M. (2011). Post-error adjustments. Front. Psychol. 2:233. 10.3389/fpsyg.2011.00233 21954390 PMC3173829

[B16] Dayan P. (2009). Goal-directed control and its antipodes. Neural Netw. 22, 213–219. 10.1016/j.neunet.2009.03.004 19362448

[B17] de Ridder D. T. de Boer B. J. Lugtig P. Bakker A. B. van Hooft E. A. (2011). Not doing bad things is not equivalent to doing the right thing: distinguishing between inhibitory and initiatory self-control. Personal. Individual Differ. 50, 1006–1011. 10.1016/j.paid.2011.01.015

[B18] de Ridder D. T. Lensvelt-Mulders G. Finkenauer C. Stok F. M. Baumeister R. F. (2012). Taking stock of self-control: a meta-analysis of how trait self-control relates to a wide range of behaviors. Personal. Soc. Psychol. Rev. 16, 76–99. 10.1177/1088868311418749 21878607

[B19] Debener S. Ullsperger M. Siegel M. Fiehler K. Von Cramon D. Y. Engel A. K. (2005). Trial-by-trial coupling of concurrent electroencephalogram and functional magnetic resonance imaging identifies the dynamics of performance monitoring. J. Neurosci. 25, 11730–11737. 10.1523/JNEUROSCI.3286-05.2005 16354931 PMC6726024

[B20] Delorme A. Makeig S. (2004). EEGLAB: an open source toolbox for analysis of single-trial EEG dynamics including independent component analysis. J. Neurosci. Methods 134, 9–21. 10.1016/j.jneumeth.2003.10.009 15102499

[B21] Dignath D. Eder A. B. Steinhauser M. Kiesel A. (2020). Conflict monitoring and the affective-signaling hypothesis—an integrative review. Psychonomic Bullet. Rev. 9:1–24. 10.3758/s13423-019-01668-9 31898269

[B22] Duckworth A. L. Milkman K. L. Laibson D. (2018). Beyond willpower: strategies for reducing failures of self-control. Psychol. Sci. Public Interest 19, 102–129. 10.1177/1529100618821893 30760176

[B23] Efron B. (1983). Estimating the error rate of a prediction rule: improvement on cross-validation. J. Am. Statist. Assoc. 78, 316–331. 10.1080/01621459.1983.10477973

[B24] Endrass T. Klawohn J. Gruetzmann R. Ischebeck M. Kathmann N. (2012). Response-related negativities following correct and incorrect responses: evidence from a temporospatial principal component analysis. Psychophysiology 49, 733–743. 10.1111/j.1469-8986.2012.01365.x 22417070

[B25] Endrass T. Reuter B. Kathmann N. (2007). ERP correlates of conscious error recognition: aware and unaware errors in an antisaccade task. Eur. J. Neurosci. 26, 1714–1720. 10.1111/j.1460-9568.2007.05785.x 17880402

[B26] Endrass T. Schuermann B. Kaufmann C. Spielberg R. Kniesche R. Kathmann N. (2010). Performance monitoring and error significance in patients with obsessive-compulsive disorder. Biol. Psychol. 84, 257–263. 10.1016/j.biopsycho.2010.02.002 20152879

[B27] Endrass T. Ullsperger M. (2014). Specificity of performance monitoring changes in obsessive-compulsive disorder. Neurosci. Biobehav. Rev. 46, 124–138. 10.1016/j.neubiorev.2014.03.024 24747486

[B28] Engebretsen S. Bohlin J. (2019). Statistical predictions with glmnet. Clin. Epigenet. 11, 1–3. 10.1186/s13148-019-0730-1 31443682 PMC6708235

[B29] Eriksen B. A. Eriksen C. W. (1974). Effects of noise letters upon the identification of a target letter in a nonsearch task. Perception Psychophys. 16, 143–149. 10.3758/BF03203267

[B30] Euser A. S. Evans B. E. Greaves-Lord K. Huizink A. C. Franken I. H. (2013). Diminished error-related brain activity as a promising endophenotype for substance-use disorders: evidence from high-risk offspring. Addict. Biol. 18, 970–984. 10.1111/adb.12002 23145495

[B31] Falkenstein M. Hohnsbein J. Hoormann J. Blanke L. (1991). Effects of crossmodal divided attention on late ERP components. II. Error processing in choice reaction tasks. Electroencephalogr. Clin. Neurophysiol. 78, 447–455. 10.1016/0013-4694(91)90062-9 1712280

[B32] Fan J. Li R. (2001). Variable selection *via* nonconcave penalized likelihood and its oracle properties. J. Am. Statist. Assoc. 96, 1348–1360. 10.1198/016214501753382273

[B33] Fan J. Peng H. (2004). Nonconcave penalized likelihood with a diverging number of parameters. Annal. Statist. 32, 928–961. 10.1214/009053604000000256

[B34] Fischer A. G. Ullsperger M. (2013). Real and fictive outcomes are processed differently but converge on a common adaptive mechanism. Neuron 79, 1243–1255. 10.1016/j.neuron.2013.07.006 24050408

[B35] Ford J. M. (1999). Schizophrenia: the broken P300 and beyond. Psychophysiology 36, 667–682. 10.1111/1469-8986.3660667 10554581

[B36] Friedman J. Hastie T. Tibshirani R. (2010). Regularization paths for generalized linear models via coordinate descent. J. Stat. Softw. 33:1. 10.18637/jss.v033.i01 20808728 PMC2929880

[B37] Fujita K. (2011). On conceptualizing self-control as more than the effortful inhibition of impulses. Personal. Soc. Psychol. Rev. 15, 352–366. 10.1177/1088868311411165 21685152

[B38] Gehring W. J. Goss B. Coles M. G. Meyer D. E. Donchin E. (1993). A neural system for error detection and compensation. Psychol. Sci. 4, 385–390. 10.1111/j.1467-9280.1993.tb00586.x

[B39] Gehring W. J. Goss B. Coles M. G. Meyer D. E. Donchin E. (2018). The error-related negativity. Perspect. Psychol. Sci. 13, 200–204. 10.1177/1745691617715310 29592655

[B40] Gillan C. Fineberg N. Robbins T. (2017). A trans-diagnostic perspective on obsessive-compulsive disorder. Psychol. Med. 47, 1528–1548. 10.1017/S0033291716002786 28343453 PMC5964477

[B41] Gillebaart M. (2018). The “operational” definition of self-control. Front. Psychol. 9:1231. 10.3389/fpsyg.2018.01231 30072939 PMC6058080

[B42] Gollwitzer P. M. (1999). Implementation intentions: strong effects of simple plans. Am. Psychol. 54:493 10.1037/0003-066X.54.7.493

[B43] Goschke T. (2014). Dysfunctions of decision-making and cognitive control as transdiagnostic mechanisms of mental disorders: advances, gaps, and needs in current research. Int. J. Methods Psychiatr. Res. 23, 41–57. 10.1002/mpr.1410 24375535 PMC6878557

[B44] Grisetto F. Delevoye-Turrell Y. N. Roger C. (2019). Efficient but less active monitoring system in individuals with high aggressive predispositions. Int. J. Psychophysiol. 146, 125–132. 10.1016/j.ijpsycho.2019.10.006 31669317

[B45] Grömping U. (2009). Variable importance assessment in regression: linear regression versus random forest. Am. Statist. 63, 308–319. 10.1198/tast.2009.08199

[B46] Grosenick L. Klingenberg B. Katovich K. Knutson B. Taylor J. E. (2013). Interpretable whole-brain prediction analysis with GraphNet. NeuroImage 72, 304–321. 10.1016/j.neuroimage.2012.12.062 23298747

[B47] Grützmann R. Riesel A. Klawohn J. Kathmann N. Endrass T. (2014). Complementary modulation of N 2 and CRN by conflict frequency. Psychophysiology 51, 761–772. 10.1111/psyp.12222 24735386

[B48] Hare T. A. Camerer C. F. Rangel A. (2009). Self-control in decision-making involves modulation of the vmPFC valuation system. Science 324, 646–648. 10.1126/science.1168450 19407204

[B49] Harrell F. E. (2015). Regression Modeling Strategies: With Applications to Linear Models, Logistic and Ordinal Regression, and Survival Analysis. Switzerland: Springer 10.1007/978-3-319-19425-7

[B50] Heatherton T. F. Wagner D. D. (2011). Cognitive neuroscience of self-regulation failure. Trends Cogn. Sci. 15, 132–139. 10.1016/j.tics.2010.12.005 21273114 PMC3062191

[B51] Hedge C. Powell G. Sumner P. (2018). The reliability paradox: why robust cognitive tasks do not produce reliable individual differences. Behav. Res. Methods 50, 1166–1186. 10.3758/s13428-017-0935-1 28726177 PMC5990556

[B52] Hofmann W. Baumeister R. F. Förster G. Vohs K. D. (2012a). Everyday temptations: an experience sampling study of desire, conflict, and self-control. J. Personal. Soc. Psychol. 102:1318. 10.1037/a0026545 22149456

[B53] Hofmann W. Kotabe H. (2012). A general model of preventive and interventive self-control. Soc. Personal. Psychol. Compass 6, 707–722. 10.1111/j.1751-9004.2012.00461.x

[B54] Hofmann W. Schmeichel B. J. Baddeley A. D. (2012b). Executive functions and self-regulation. Trends Cogn. Sci. 16, 174–180. 10.1016/j.tics.2012.01.006 22336729

[B55] Holroyd C. B. Coles M. G. (2002). The neural basis of human error processing: reinforcement learning, dopamine, and the error-related negativity. Psychol. Rev. 109:679. 10.1037/0033-295X.109.4.679 12374324

[B56] Huster R. J. Eichele T. Enriquez-Geppert S. Wollbrink A. Kugel H. Konrad C.. (2011). Multimodal imaging of functional networks and event-related potentials in performance monitoring. Neuroimage 56, 1588–1597. 10.1016/j.neuroimage.2011.03.039 21421060

[B57] Inzlicht M. Gutsell J. N. (2007). Running on empty: neural signals for self-control failure. Psychol. Sci. 18, 933–937. 10.1111/j.1467-9280.2007.02004.x 17958704

[B58] Inzlicht M. Werner K. M. Briskin J. Roberts B. (2020). Integrating models of self-regulation. Ann. Rev. 72:105721. 10.31234/osf.io/dpjye 33017559

[B59] James G. Witten D. Hastie T. Tibshirani R. (2013). An Introduction to Statistical Learning. New York, NY: Springer 10.1007/978-1-4614-7138-7

[B60] Jansen M. de Bruijn E. (2020). Mistakes that matter: an event-related potential study on obsessive-compulsive symptoms and social performance monitoring in different responsibility contexts. Cogn. Affect. Behav. Neurosci. 20, 684–697. 10.3758/s13415-020-00796-3 32372323 PMC7394925

[B61] Keil J. Weisz N. Paul-Jordanov I. Wienbruch C. (2010). Localization of the magnetic equivalent of the ERN and induced oscillatory brain activity. Neuroimage 51, 404–411. 10.1016/j.neuroimage.2010.02.003 20149884

[B62] Kerns J. G. Cohen J. D. MacDonald A. W. Cho R. Y. Stenger V. A. Carter C. S. (2004). Anterior cingulate conflict monitoring and adjustments in control. Science 303, 1023–1026. 10.1126/science.1089910 14963333

[B63] Kopp B. Rist F. Mattler U. (1996). N200 in the flanker task as a neurobehavioral tool for investigating executive control. Psychophysiology 33, 282–294. 10.1111/j.1469-8986.1996.tb00425.x 8936397

[B64] Krönke K.-M. Wolff M. Mohr H. Kräplin A. Smolka M. N. Bühringer G.. (2018). Monitor yourself! Deficient error-related brain activity predicts real-life self-control failures. Cogn. Affect. Behav. Neurosci. 18, 622–637. 10.3758/s13415-018-0593-5 29654477

[B65] Krönke K.-M. Wolff M. Mohr H. Kräplin A. Smolka M. N. Bühringer G.. (2020). Predicting real-life self-control from brain activity encoding the value of anticipated future outcomes. Psychol. Sci. 31, 268–279. 10.1177/0956797619896357 32024421

[B66] Kuhn M. (2008). Building predictive models in R using the caret package. J. Stat. Softw. 28, 1–26. 10.18637/jss.v028.i0527774042

[B67] Kuhn M. Wing J. Weston S. Williams A. Keefer C. Engelhardt A. (2020). Package “caret”: Classification and Regression Training. Available online at: https://cran.r-project.org/web/packages/caret/caret.pdf (accessed May 25, 2020).

[B68] Lopez R. B. Hofmann W. Wagner D. D. Kelley W. M. Heatherton T. F. (2014). Neural predictors of giving in to temptation in daily life. Psychol. Sci. 25, 1337–1344. 10.1177/0956797614531492 24789842 PMC4214912

[B69] Luijten M. Machielsen M. W. Veltman D. J. Hester R. de Haan L. Franken I. H. (2014). Systematic review of ERP and fMRI studies investigating inhibitory control and error processing in people with substance dependence and behavioural addictions. J. Psychiatry Neurosci. 39, 149–169. 10.1503/jpn.130052 24359877 PMC3997601

[B70] Mair P. Wilcox R. (2019). Robust statistical methods in R using the WRS2 package. Behav. Res. Methods 52, 464–488. 10.3758/s13428-019-01246-w 31152384

[B71] Meyer A. Hajcak G. (2019). A review examining the relationship between individual differences in the error-related negativity and cognitive control. Int. J. Psychophysiol. 144, 7–13. 10.1016/j.ijpsycho.2019.07.005 31362030

[B72] Miyake A. Friedman N. P. (2012). The nature and organization of individual differences in executive functions: four general conclusions. Curr. Direct. Psychol. Sci. 21, 8–14. 10.1177/0963721411429458 22773897 PMC3388901

[B73] Muraven M. Slessareva E. (2003). Mechanisms of self-control failure: motivation and limited resources. Personal. Soc. Psychol. Bullet. 29, 894–906. 10.1177/0146167203029007008 15018677

[B74] Myrseth K. O. R. Fishbach A. (2009). Self-control: a function of knowing when and how to exercise restraint. Curr. Direct. Psychol. Sci. 18, 247–252. 10.1111/j.1467-8721.2009.01645.x

[B75] Nee D. E. Kastner S. Brown J. W. (2011). Functional heterogeneity of conflict, error, task-switching, and unexpectedness effects within medial prefrontal cortex. Neuroimage 54, 528–540. 10.1016/j.neuroimage.2010.08.027 20728547 PMC2962721

[B76] Notebaert W. Houtman F. Van Opstal F. Gevers W. Fias W. Verguts T. (2009). Post-error slowing: an orienting account. Cognition 111, 275–279. 10.1016/j.cognition.2009.02.002 19285310

[B77] Olvet D. M. Hajcak G. (2009). The stability of error-related brain activity with increasing trials. Psychophysiology 46, 957–961. 10.1111/j.1469-8986.2009.00848.x 19558398

[B78] Pasion R. Barbosa F. (2019). ERN as a transdiagnostic marker of the internalizing-externalizing spectrum: a dissociable meta-analytic effect. Neurosci. Biobehav. Rev. 103, 133–149. 10.1016/j.neubiorev2019.06.013 31220503

[B79] R Core Team (2020). R: A Language and Environment for Statistical Computing. Vienna: R Foundation for Statistical Computing Available online at: https://www.R-project.org/

[B80] Ram N. Brinberg M. Pincus A. L. Conroy D. E. (2017). The questionable ecological validity of ecological momentary assessment: considerations for design and analysis. Res. Human Dev. 14, 253–270. 10.1080/15427609.2017.1340052 30613195 PMC6317726

[B81] Riesel A. Kathmann N. Endrass T. (2014). Overactive performance monitoring in obsessive–compulsive disorder is independent of symptom expression. Eur. Archiv. Psychiatry Clin. Neurosci. 264, 707–717. 10.1007/s00406-014-0499-3 24676800

[B82] Riesel A. Weinberg A. Endrass T. Meyer A. Hajcak G. (2013). The ERN is the ERN is the ERN? Convergent validity of error-related brain activity across different tasks. Biol. Psychol. 93, 377–385. 10.1016/j.biopsycho.2013.04.007 23607999

[B83] Robbins T. W. Gillan C. M. Smith D. G. de Wit S. Ersche K. D. (2012). Neurocognitive endophenotypes of impulsivity and compulsivity: towards dimensional psychiatry. Trends Cogn. Sci. 16, 81–91. 10.1016/j.tics.2011.11.009 22155014

[B84] Rushworth M. Walton M. E. Kennerley S. W. Bannerman D. (2004). Action sets and decisions in the medial frontal cortex. Trends Cogn. Sci. 8, 410–417. 10.1016/j.tics.2004.07.009 15350242

[B85] Rushworth M. F. (2008). Intention, choice, and the medial frontal cortex. Annal. NY Acad. Sci. 1124, 181–207. 10.1196/annals.1440.014 18400931

[B86] Saunders B. Jentzsch I. (2012). False external feedback modulates posterror slowing and the f-P300: implications for theories of posterror adjustment. Psychonomic Bullet. Rev. 19, 1210–1216. 10.3758/s13423-012-0314-y 22987148

[B87] Schmidt J. R. (2019). Evidence against conflict monitoring and adaptation: an updated review. Psychonomic Bullet. Rev. 26, 753–771. 10.3758/s13423-018-1520-z 30511233

[B88] Shenhav A. Botvinick M. M. Cohen J. D. (2013). The expected value of control: an integrative theory of anterior cingulate cortex function. Neuron 79, 217–240. 10.1016/j.neuron.2013.07.007 23889930 PMC3767969

[B89] Steinhauser M. Yeung N. (2010). Decision processes in human performance monitoring. J. Neurosci. 30, 15643–15653. 10.1523/JNEUROSCI.1899-10.2010 21084620 PMC3073548

[B90] The MathWorks Inc (2018). “MATLAB”. version 9.5.0.944444 (R2018b) ed. Natick, MA.

[B91] Troller-Renfree S. Zeanah C. H. Nelson C. A. Fox N. A. (2018). Neural and cognitive factors influencing the emergence of psychopathology: insights from the Bucharest early intervention project. Child Dev. Perspect. 12, 28–33. 10.1111/cdep.12251 29531577 PMC5844478

[B92] Ullsperger M. Danielmeier C. Jocham G. (2014a). Neurophysiology of performance monitoring and adaptive behavior. Physiol. Rev. 94, 35–79. 10.1152/physrev.00041.2012 24382883

[B93] Ullsperger M. Fischer A. G. Nigbur R. Endrass T. (2014b). Neural mechanisms and temporal dynamics of performance monitoring. Trends Cogn. Sci. 18, 259–267. 10.1016/j.tics.2014.02.009 24656460

[B94] Ullsperger M. Harsay H. A. Wessel J. R. Ridderinkhof K. R. (2010). Conscious perception of errors and its relation to the anterior insula. Brain Struct. Function 214, 629–643. 10.1007/s00429-010-0261-1 20512371 PMC2886909

[B95] Van Veen V. Carter C. S. (2002). The anterior cingulate as a conflict monitor: fMRI and ERP studies. Physiol. Behav. 77, 477–482. 10.1016/S0031-9384(02)00930-7 12526986

[B96] Venables W. N. Ripley B. D. (2002). Modern Applied Statistics With S, 4th Edn. New York, NY: Springer 10.1007/978-0-387-21706-2

[B97] Vohs K. D. Baumeister R. F. Schmeichel B. J. (2012). Motivation, personal beliefs, and limited resources all contribute to self-control. J. Exp. Soc. Psychol. 48, 943–947. 10.1016/j.jesp.2012.03.002

[B98] Weinberg A. Dieterich R. Riesel A. (2015). Error-related brain activity in the age of RDoC: a review of the literature. Int. J. Psychophysiol. 98, 276–299. 10.1016/j.ijpsycho.2015.02.029 25746725

[B99] Wessel J. R. Danielmeier C. Morton J. B. Ullsperger M. (2012). Surprise and error: common neuronal architecture for the processing of errors and novelty. J. Neurosci. 32, 7528–7537. 10.1523/JNEUROSCI.6352-11.2012 22649231 PMC6703591

[B100] Wessel J. R. Danielmeier C. Ullsperger M. (2011). Error awareness revisited: accumulation of multimodal evidence from central and autonomic nervous systems. J. Cogn. Neurosci. 23, 3021–3036. 10.1162/jocn.2011.21635 21268673

[B101] Wolff M. Krönke K.-M. Venz J. Kräplin A. Bühringer G. Smolka M. N.. (2016). Action versus state orientation moderates the impact of executive functioning on real-life self-control. J. Exp. Psychol. 145:1635. 10.1037/xge0000229 27736135

[B102] Xiao N. Xu Q.-S. (2015). Multi-step adaptive elastic-net: reducing false positives in high-dimensional variable selection. J. Statist. Comput. Simul. 85, 3755–3765. 10.1080/00949655.2015.1016944

[B103] Zou H. (2006). The adaptive lasso and its oracle properties. J. Am. Statist. Assoc. 101, 1418–1429. 10.1198/016214506000000735

[B104] Zou H. Hastie T. (2005). Regularization and variable selection *via* the elastic net. J. Royal Statist. Soc. 67, 301–320. 10.1111/j.1467-9868.2005.00503.x

[B105] Zou H. Zhang H. H. (2009). On the adaptive elastic-net with a diverging number of parameters. Annal. Statist. 37:1733. 10.1214/08-AOS625 20445770 PMC2864037

