# Supplementary material for: The Error-Related Negativity Predicts Self-Control Failures in Daily Life
Source: Front Hum Neurosci. 2021 Jan 27;14:614979. doi: 10.3389/fnhum.2020.614979 (PMC7873054; doi:10.3389/fnhum.2020.614979)
Supplement: Supplementary file 1 [file Table_1.docx]

Supplementary Material

# Supplementary Data

*Additional EEG analysis by incentive context*. Mean ERN amplitude in a time window of 20 ms around the individual ERN peak, averaged at electrodes FCz, Fz, F1 and F2, predicted SCFs within a GLM based on negative binomial distribution for both the gain (*β* = .053, *z* = 2.252, *p* < .05) and the loss avoidance context (*β* = .061, *z* = 2.451, *p* < .05).

*Additional EEG analysis for difference scores and CRN*. Mean ERN amplitude in a time window of 20 ms around the individual peak, averaged at electrodes FCz, Fz, F1 and F2 were determined. Difference scores of the within-subject averaged response-locked EEG for incongruent error minus incongruent correct trials predicted SCFs within a GLM based on negative binomial distribution (*β* = .039, *z* = 2.001, *p* < .05), the CRN did not (*β* = -.018, *z* = -0.641, *p* = .52).

# Supplementary Figures and Tables

## Supplementary Figures

| 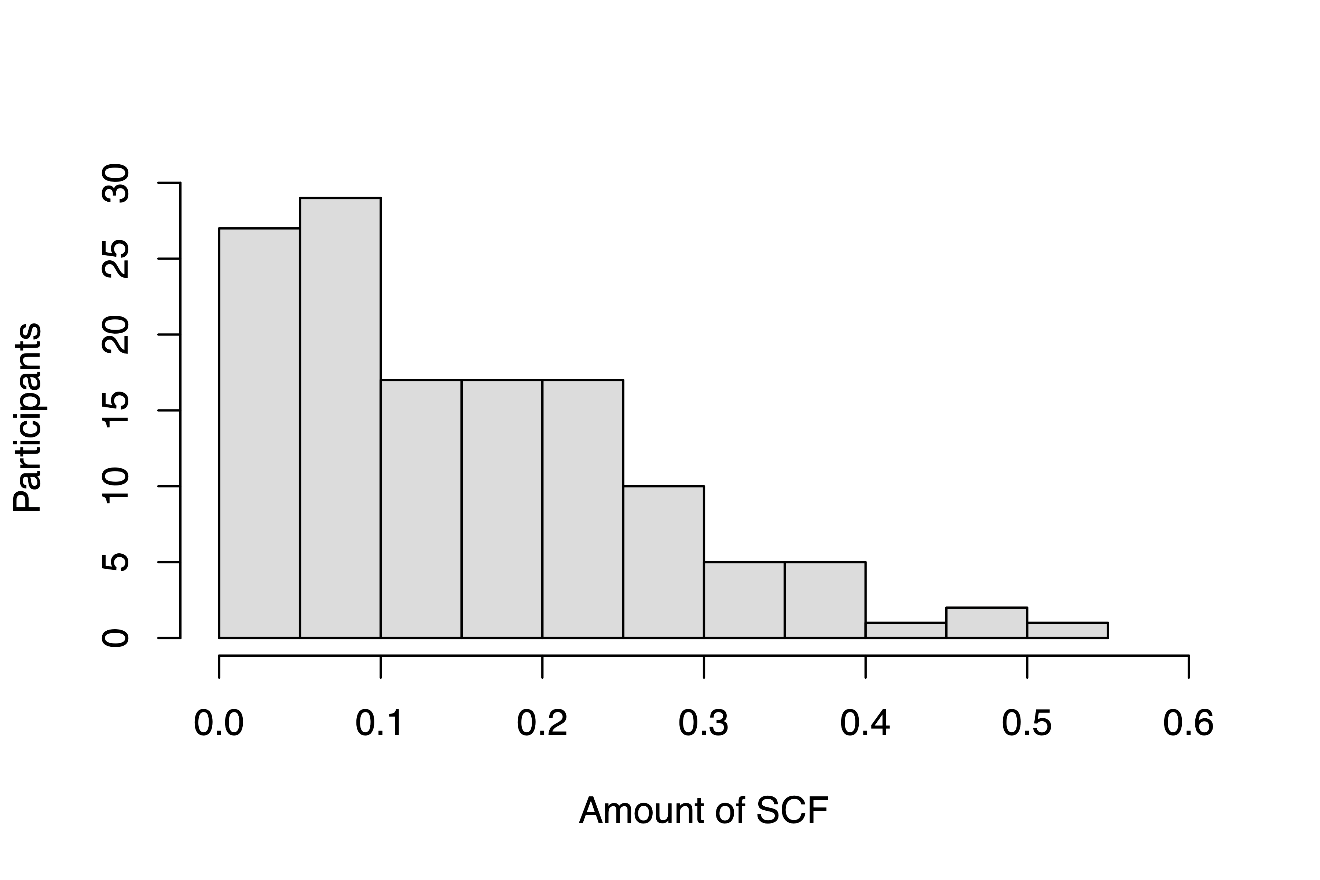 |
| --- |
| **Supplementary Figure 1 \|** Distribution of the number of self-control failures (SCFs) across participants as reported in ecological momentary assessment. |


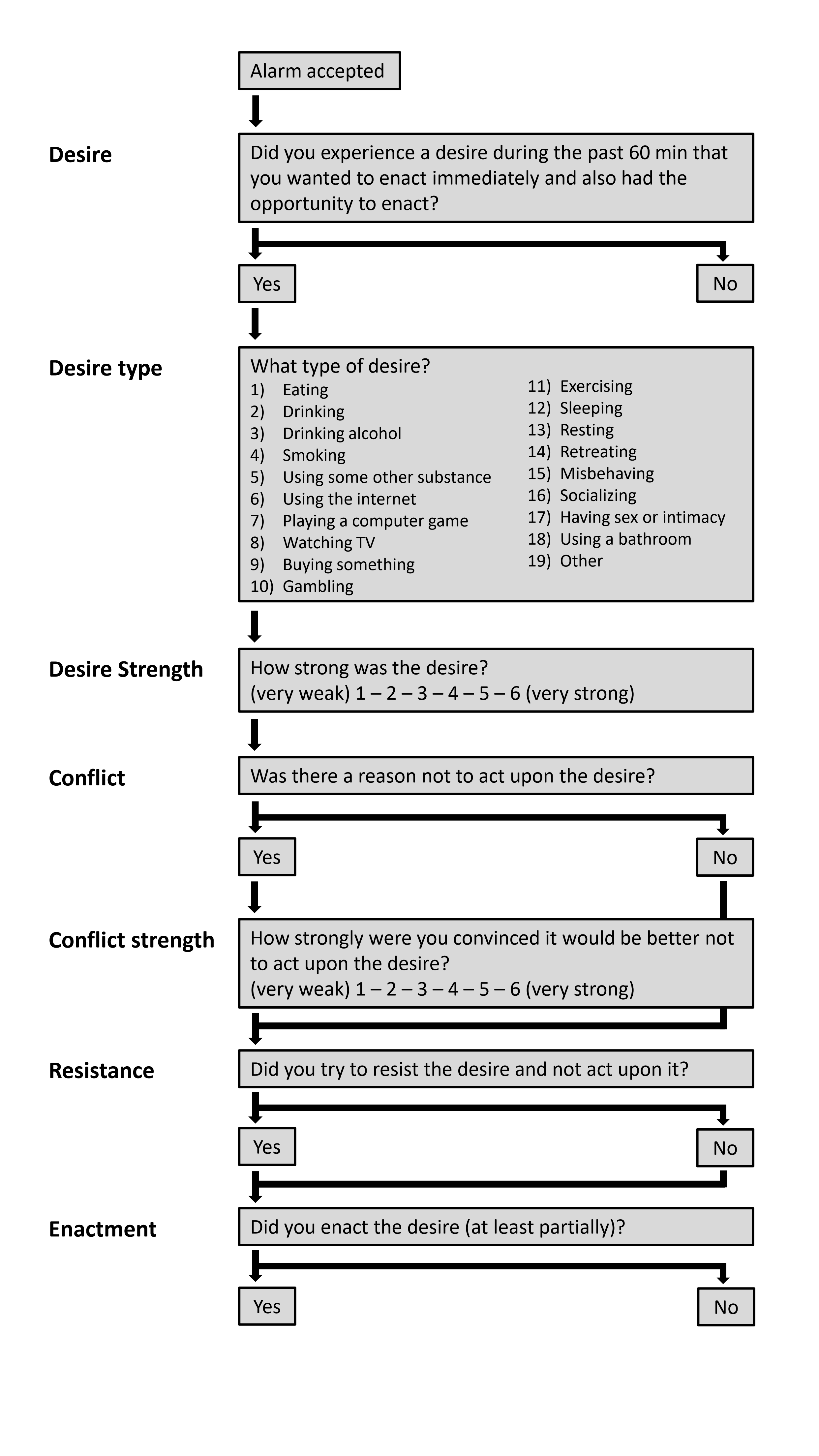


**Supplementary Figure 2 |** Questionnaire of ecological momentary assessment of self-control in daily life.
